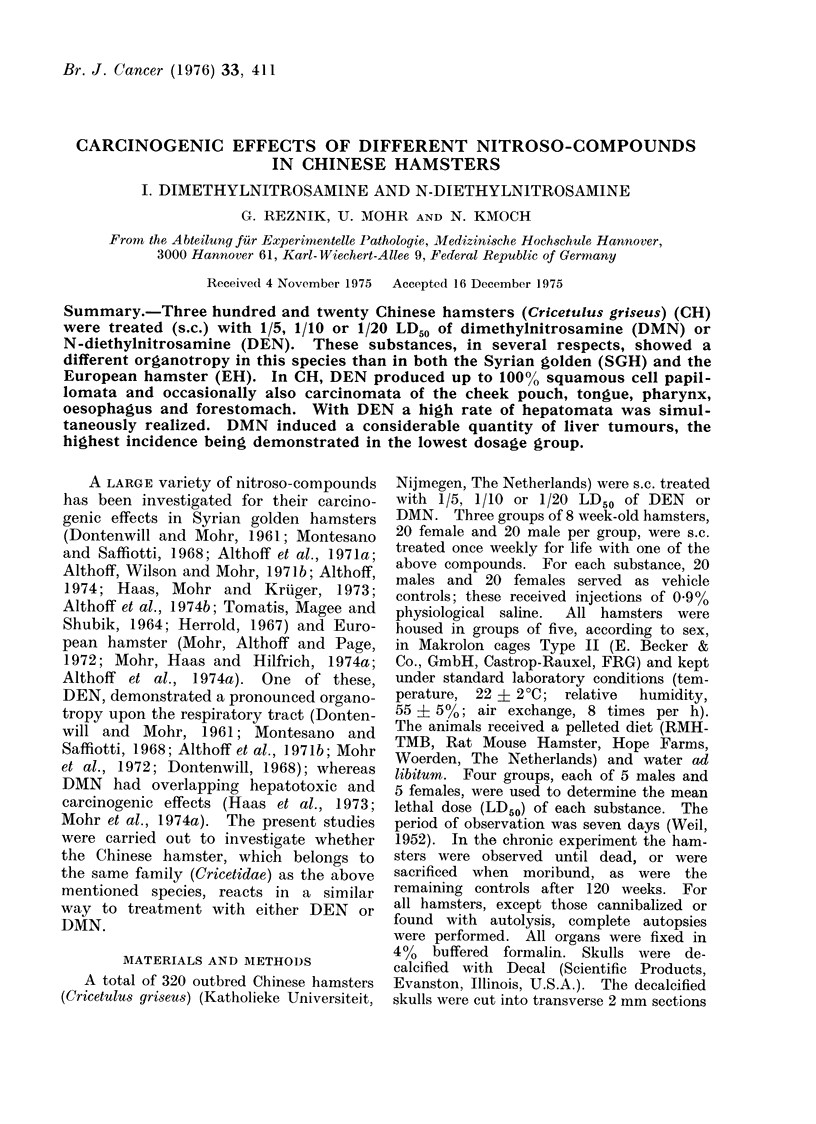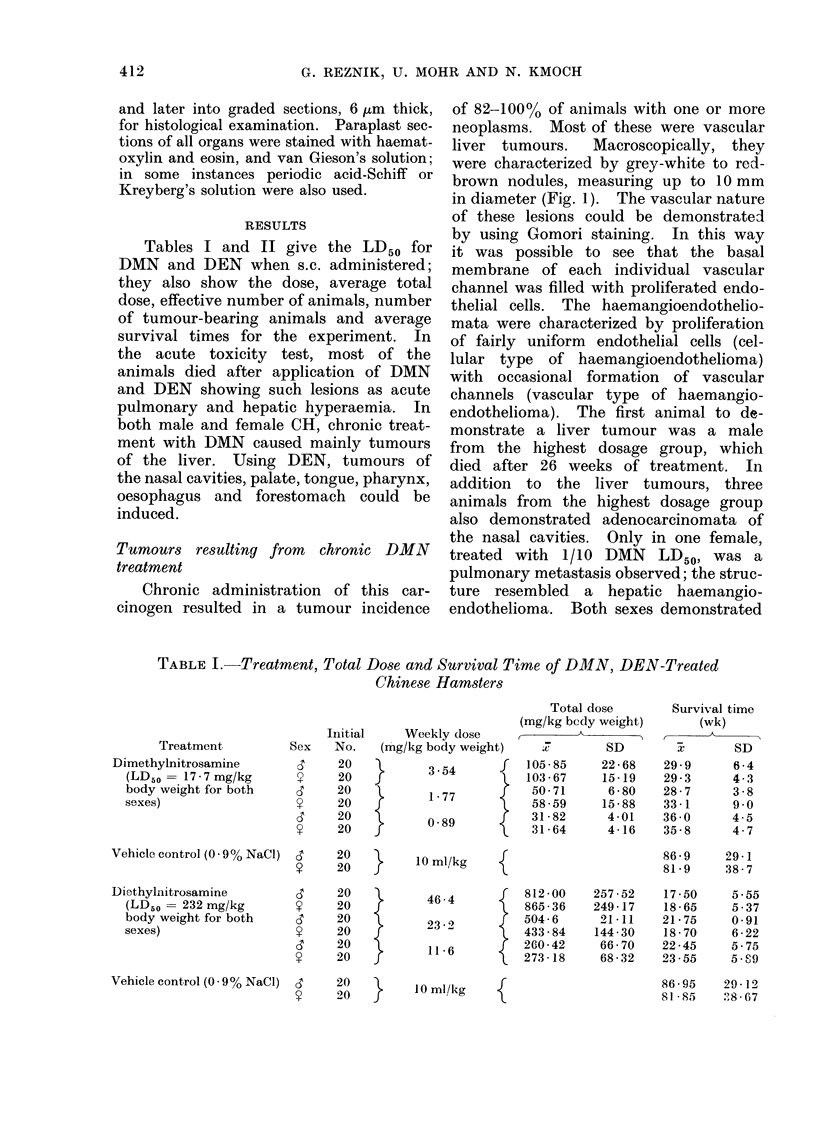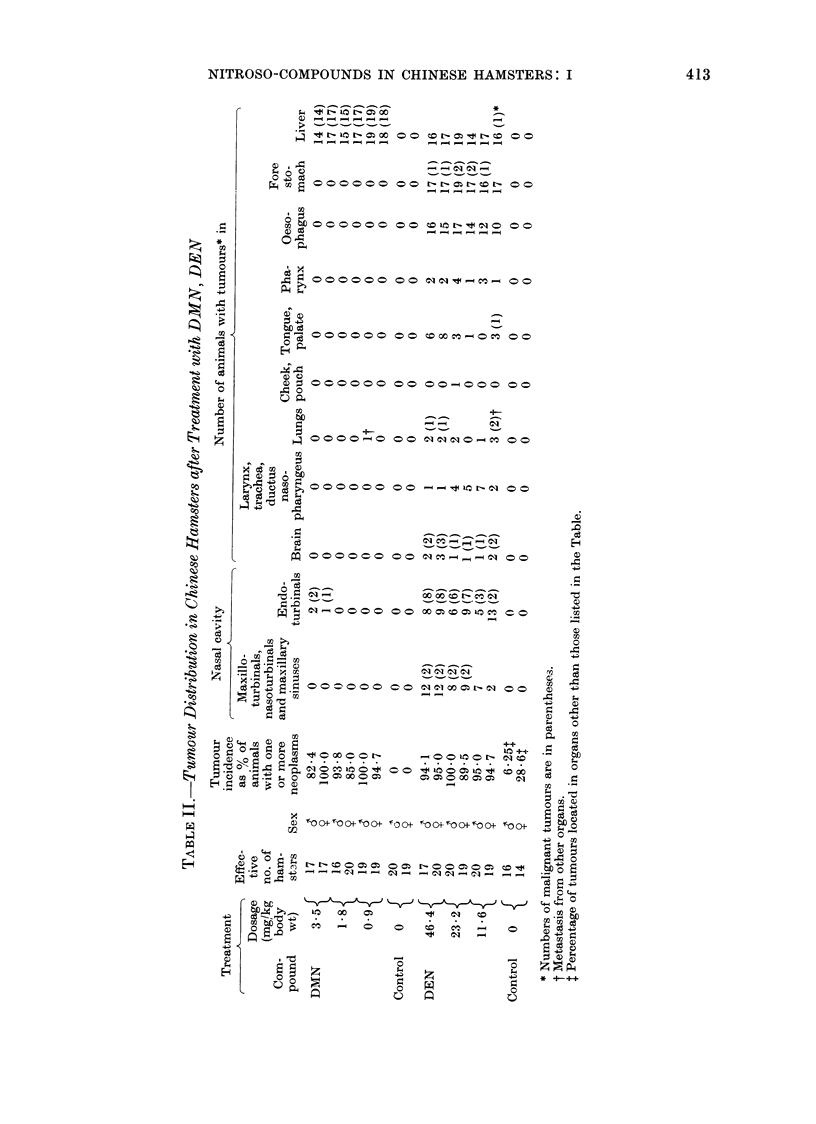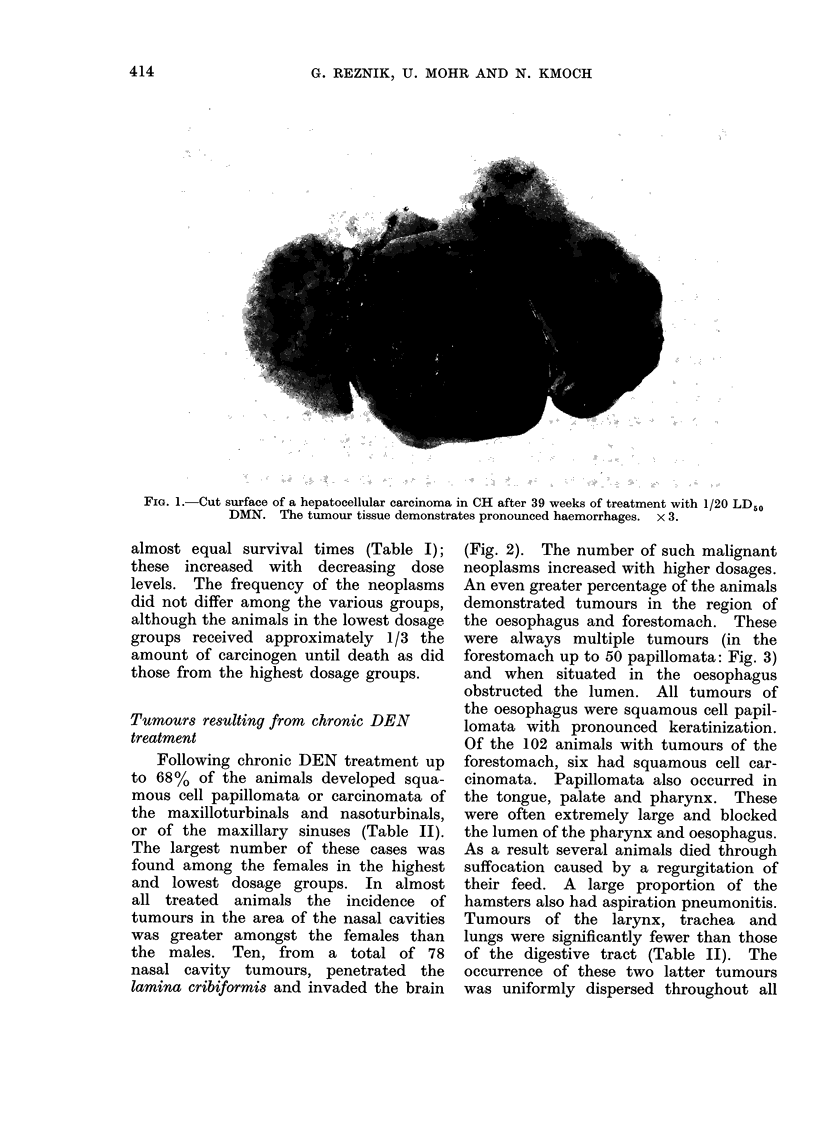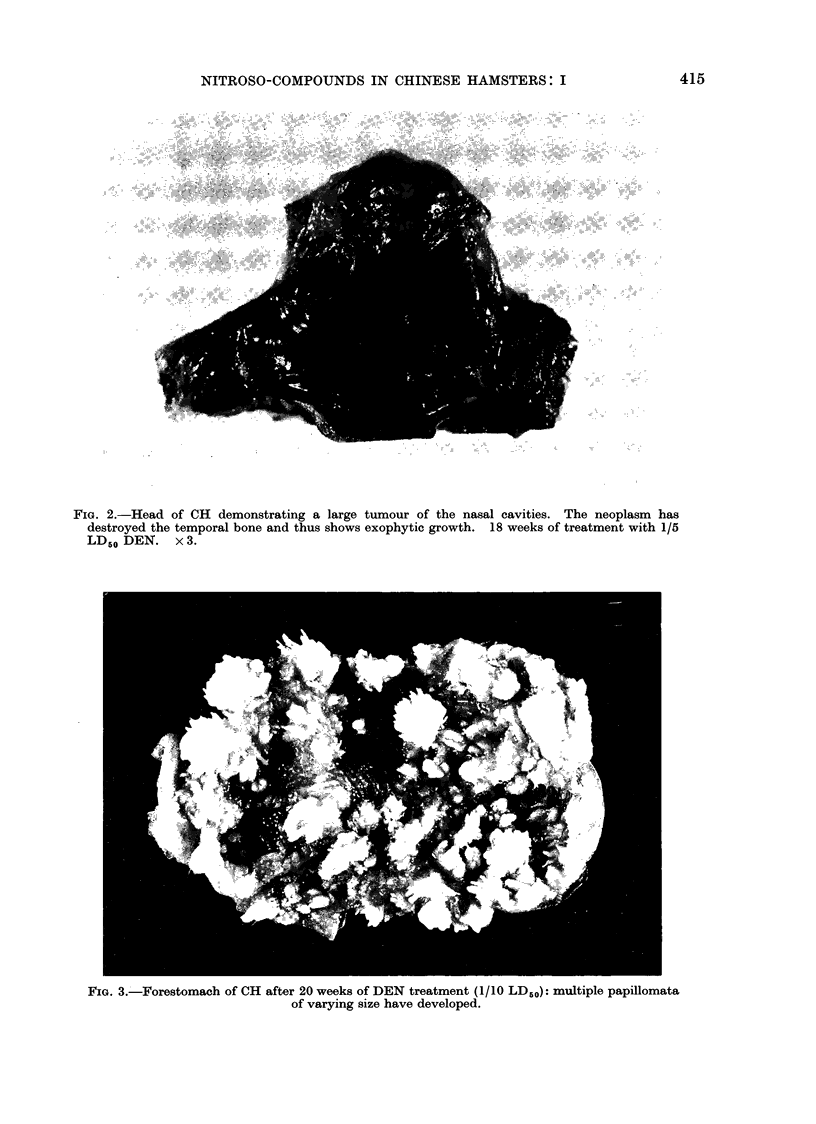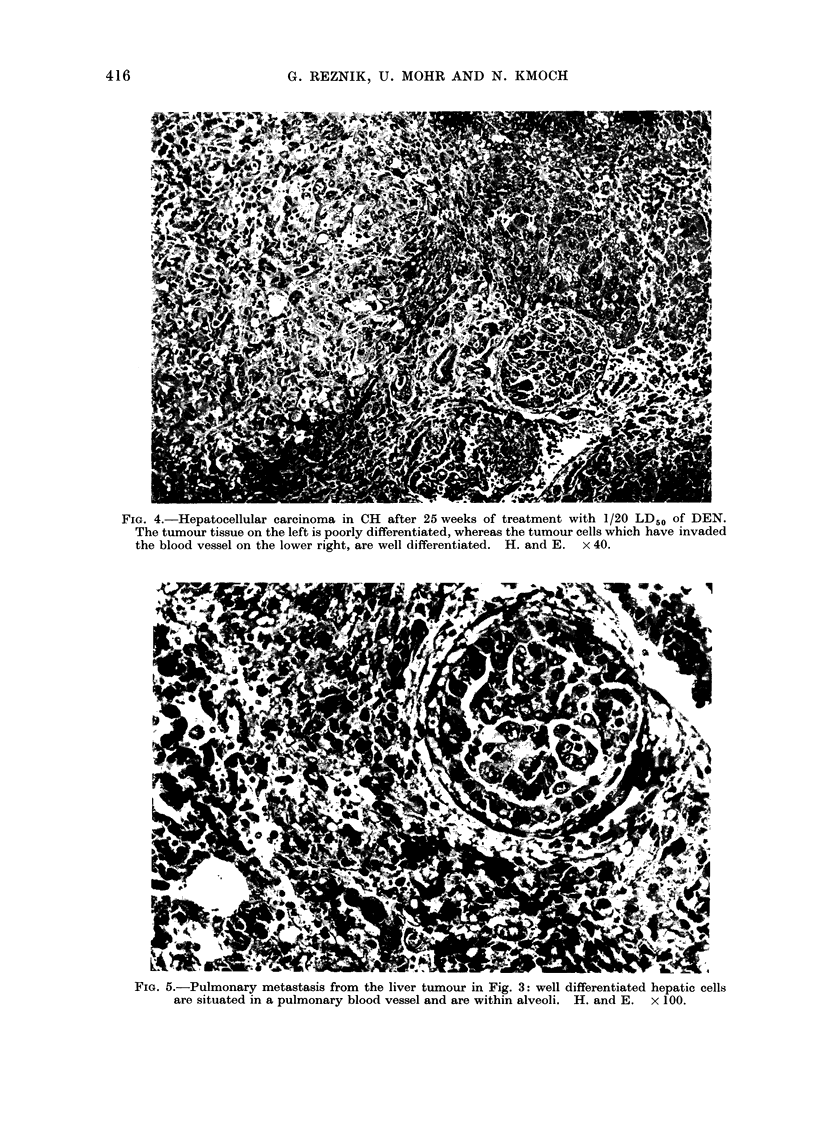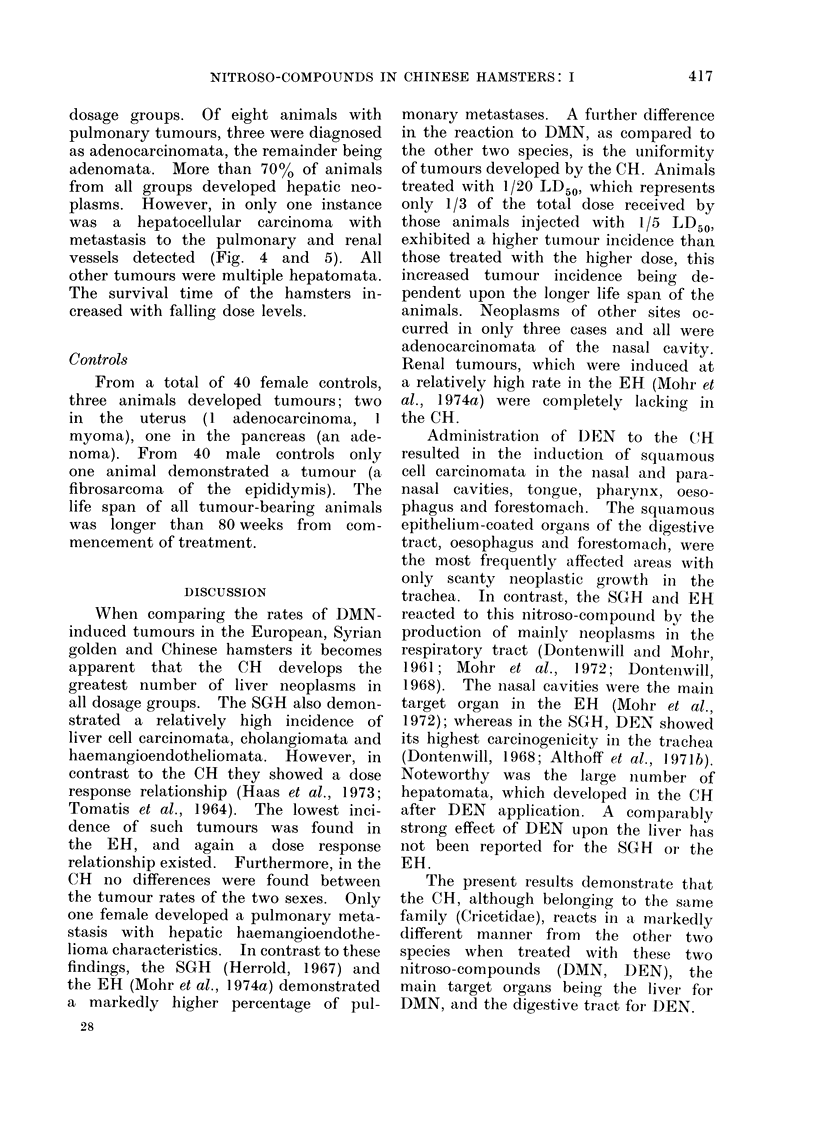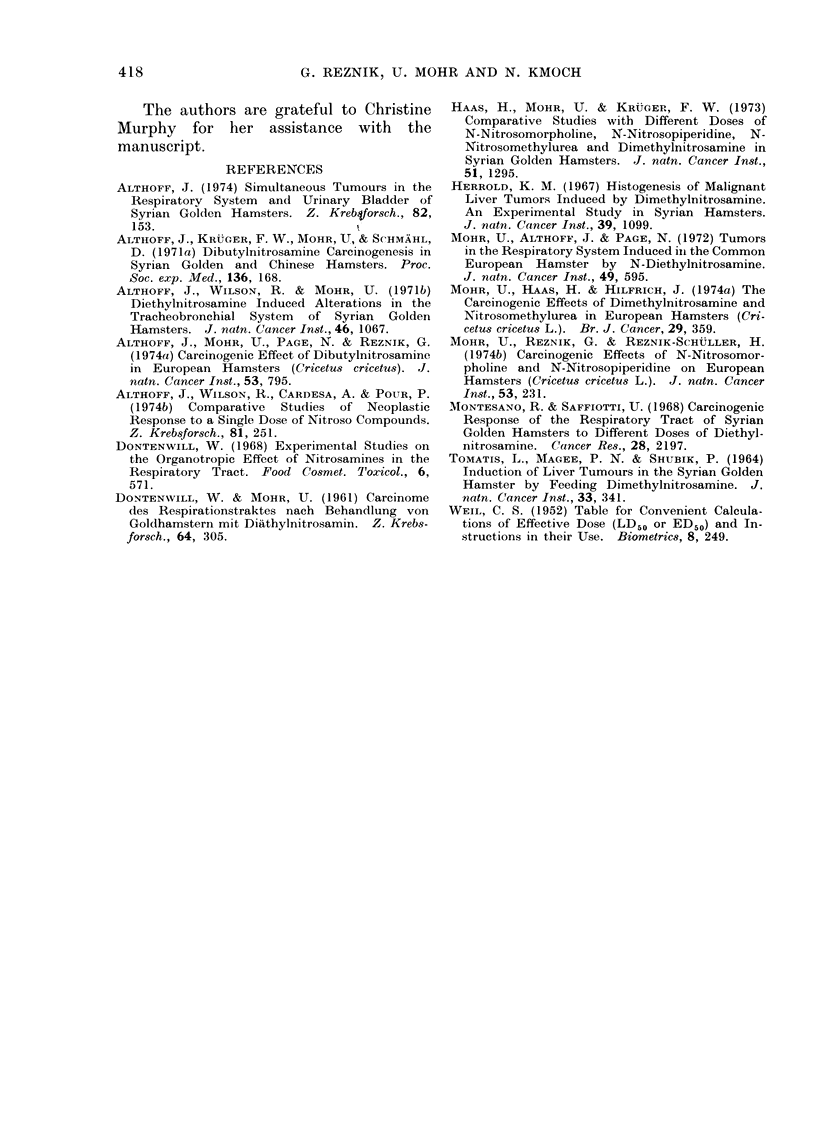# Carcinogenic effects of different nitroso-compounds in Chinese hamsters. I. Dimethylnitrosamine and N-diethylnitrosamine.

**DOI:** 10.1038/bjc.1976.66

**Published:** 1976-04

**Authors:** G. Reznik, U. Mohr, N. Kmoch

## Abstract

**Images:**


*Br. J. Cancer* (1976) **33**, 411

### CARCINOGENIC EFFECTS OF DIFFERENT NITROSO-COMPOUNDS IN CHINESE HAMSTERS

### I. DIMETHYLNITROSAMINE AND N-DIETHYLNITROSAMINE

G. REZNIK, U. MOHR AND N. KMOCH

*From the Abteilung für Experimentelle Pathologie, Medizinische Hochschule Hannover, 3000 Hannover 61, Karl-Wiechert-Allee 9, Federal Republic of Germany*

Received 4 November 1975 Accepted 16 December 1975

**Summary.—Three hundred and twenty Chinese hamsters (*Cricetulus griseus*) (CH) were treated (s.c.) with 1/5, 1/10 or 1/20 $LD_{50}$ of dimethylnitrosamine (DMN) or N-diethylnitrosamine (DEN). These substances, in several respects, showed a different organotropy in this species than in both the Syrian golden (SGH) and the European hamster (EH). In CH, DEN produced up to 100% squamous cell papillomata and occasionally also carcinomata of the cheek pouch, tongue, pharynx, oesophagus and forestomach. With DEN a high rate of hepatomata was simultaneously realized. DMN induced a considerable quantity of liver tumours, the highest incidence being demonstrated in the lowest dosage group.**

A LARGE variety of nitroso-compounds has been investigated for their carcinogenic effects in Syrian golden hamsters (Dontenwill and Mohr, 1961; Montesano and Saffiotti, 1968; Althoff *et al.*, 1971*a*; Althoff, Wilson and Mohr, 1971*b*; Althoff, 1974; Haas, Mohr and Krüger, 1973; Althoff *et al.*, 1974*b*; Tomatis, Magee and Shubik, 1964; Herrold, 1967) and European hamster (Mohr, Althoff and Page, 1972; Mohr, Haas and Hilfrich, 1974*a*; Althoff *et al.*, 1974*a*). One of these, DEN, demonstrated a pronounced organotropy upon the respiratory tract (Dontenwill and Mohr, 1961; Montesano and Saffiotti, 1968; Althoff *et al.*, 1971*b*; Mohr *et al.*, 1972; Dontenwill, 1968); whereas DMN had overlapping hepatotoxic and carcinogenic effects (Haas *et al.*, 1973; Mohr *et al.*, 1974*a*). The present studies were carried out to investigate whether the Chinese hamster, which belongs to the same family (*Cricetidae*) as the above mentioned species, reacts in a similar way to treatment with either DEN or DMN.

### MATERIALS AND METHODS

A total of 320 outbred Chinese hamsters (*Cricetulus griseus*) (Katholieke Universiteit, Nijmegen, The Netherlands) were s.c. treated with 1/5, 1/10 or 1/20 $LD_{50}$ of DEN or DMN. Three groups of 8 week-old hamsters, 20 female and 20 male per group, were s.c. treated once weekly for life with one of the above compounds. For each substance, 20 males and 20 females served as vehicle controls; these received injections of 0·9% physiological saline. All hamsters were housed in groups of five, according to sex, in Makrolon cages Type II (E. Becker & Co., GmbH, Castrop-Rauxel, FRG) and kept under standard laboratory conditions (temperature, 22 ± 2°C; relative humidity, 55 ± 5%; air exchange, 8 times per h). The animals received a pelleted diet (RMH-TMB, Rat Mouse Hamster, Hope Farms, Woerden, The Netherlands) and water *ad libitum*. Four groups, each of 5 males and 5 females, were used to determine the mean lethal dose ($LD_{50}$) of each substance. The period of observation was seven days (Weil, 1952). In the chronic experiment the hamsters were observed until dead, or were sacrificed when moribund, as were the remaining controls after 120 weeks. For all hamsters, except those cannibalized or found with autolysis, complete autopsies were performed. All organs were fixed in 4% buffered formalin. Skulls were decalcified with Decal (Scientific Products, Evanston, Illinois, U.S.A.). The decalcified skulls were cut into transverse 2 mm sections and later into graded sections, 6 $\mu$m thick, for histological examination. Paraplast sections of all organs were stained with haematoxylin and eosin, and van Gieson's solution; in some instances periodic acid-Schiff or Kreyberg's solution were also used.

### RESULTS

Tables I and II give the $LD_{50}$ for DMN and DEN when s.c. administered; they also show the dose, average total dose, effective number of animals, number of tumour-bearing animals and average survival times for the experiment. In the acute toxicity test, most of the animals died after application of DMN and DEN showing such lesions as acute pulmonary and hepatic hyperaemia. In both male and female CH, chronic treatment with DMN caused mainly tumours of the liver. Using DEN, tumours of the nasal cavities, palate, tongue, pharynx, oesophagus and forestomach could be induced.

### *Tumours resulting from chronic DMN treatment*

Chronic administration of this carcinogen resulted in a tumour incidence of 82–100% of animals with one or more neoplasms. Most of these were vascular liver tumours. Macroscopically, they were characterized by grey-white to red-brown nodules, measuring up to 10 mm in diameter (Fig. 1). The vascular nature of these lesions could be demonstrated by using Gomori staining. In this way it was possible to see that the basal membrane of each individual vascular channel was filled with proliferated endothelial cells. The haemangioendotheliomata were characterized by proliferation of fairly uniform endothelial cells (cellular type of haemangioendothelioma) with occasional formation of vascular channels (vascular type of haemangioendothelioma). The first animal to demonstrate a liver tumour was a male from the highest dosage group, which died after 26 weeks of treatment. In addition to the liver tumours, three animals from the highest dosage group also demonstrated adenocarcinomata of the nasal cavities. Only in one female, treated with 1/10 DMN $LD_{50}$, was a pulmonary metastasis observed; the structure resembled a hepatic haemangioendothelioma. Both sexes demonstrated

TABLE I.—*Treatment, Total Dose and Survival Time of DMN, DEN-Treated Chinese Hamsters*

| Treatment | Sex | Initial No. | Weekly dose (mg/kg body weight) | Total dose (mg/kg body weight) $\bar{x}$ | Total dose SD | Survival time (wk) $\bar{x}$ | Survival time SD |
|---|---|---|---|---|---|---|---|
| Dimethylnitrosamine | ♂ | 20 | 3·54 | 105·85 | 22·68 | 29·9 | 6·4 |
| ($LD_{50}$ = 17·7 mg/kg | ♀ | 20 | | 103·67 | 15·19 | 29·3 | 4·3 |
| body weight for both | ♂ | 20 | 1·77 | 50·71 | 6·80 | 28·7 | 3·8 |
| sexes) | ♀ | 20 | | 58·59 | 15·88 | 33·1 | 9·0 |
| | ♂ | 20 | 0·89 | 31·82 | 4·01 | 36·0 | 4·5 |
| | ♀ | 20 | | 31·64 | 4·16 | 35·8 | 4·7 |
| Vehicle control (0·9% NaCl) | ♂ | 20 | 10 ml/kg | | | 86·9 | 29·1 |
| | ♀ | 20 | | | | 81·9 | 38·7 |
| Diethylnitrosamine | ♂ | 20 | 46·4 | 812·00 | 257·52 | 17·50 | 5·55 |
| ($LD_{50}$ = 232 mg/kg | ♀ | 20 | | 865·36 | 249·17 | 18·65 | 5·37 |
| body weight for both | ♂ | 20 | 23·2 | 504·6 | 21·11 | 21·75 | 0·91 |
| sexes) | ♀ | 20 | | 433·84 | 144·30 | 18·70 | 6·22 |
| | ♂ | 20 | 11·6 | 260·42 | 66·70 | 22·45 | 5·75 |
| | ♀ | 20 | | 273·18 | 68·32 | 23·55 | 5·89 |
| Vehicle control (0·9% NaCl) | ♂ | 20 | 10 ml/kg | | | 86·95 | 29·12 |
| | ♀ | 20 | | | | 81·85 | 38·67 |

TABLE II.—*Tumour Distribution in Chinese Hamsters after Treatment with DMN, DEN*

| Treatment | | | | | Nasal cavity | | Number of animals with tumours* in | | | | | | | | |
|---|---|---|---|---|---|---|---|---|---|---|---|---|---|---|---|
| Compound | Dosage (mg/kg body wt) | Effective no. of hamsters | Sex | Tumour incidence as % of animals with one or more neoplasms | Maxilloturbinals, nasoturbinals and maxillary sinuses | Endoturbinals | Brain | Larynx, trachea, ductus nasopharyngeus | Lungs | Cheek pouch | Tongue, palate | Pharynx | Oesophagus | Fore stomach | Liver |
| DMN | 3·5 | 17 | ♂ | 82·4 | 0 | 2 (2) | 0 | 0 | 0 | 0 | 0 | 0 | 0 | 0 | 14 (14) |
| | | 17 | ♀ | 100·0 | 0 | 1 (1) | 0 | 0 | 0 | 0 | 0 | 0 | 0 | 0 | 17 (17) |
| | 1·8 | 16 | ♂ | 93·8 | 0 | 0 | 0 | 0 | 0 | 0 | 0 | 0 | 0 | 0 | 15 (15) |
| | | 20 | ♀ | 85·0 | 0 | 0 | 0 | 0 | 0 | 0 | 0 | 0 | 0 | 0 | 17 (17) |
| | 0·9 | 19 | ♂ | 100·0 | 0 | 0 | 0 | 0 | 1† | 0 | 0 | 0 | 0 | 0 | 19 (19) |
| | | 19 | ♀ | 94·7 | 0 | 0 | 0 | 0 | 0 | 0 | 0 | 0 | 0 | 0 | 18 (18) |
| Control | 0 | 20 | ♂ | 0 | 0 | 0 | 0 | 0 | 0 | 0 | 0 | 0 | 0 | 0 | 0 |
| | | 19 | ♀ | 0 | 0 | 0 | 0 | 0 | 0 | 0 | 0 | 0 | 0 | 0 | 0 |
| DEN | 46·4 | 17 | ♂ | 94·1 | 12 (2) | 8 (8) | 2 (2) | 1 | 2 (1) | 0 | 6 | 2 | 16 | 17 (1) | 16 |
| | | 20 | ♀ | 95·0 | 12 (2) | 9 (8) | 3 (3) | 1 | 2 (1) | 0 | 8 | 2 | 15 | 17 (1) | 17 |
| | 23·2 | 20 | ♂ | 100·0 | 8 (2) | 6 (6) | 1 (1) | 4 | 2 | 1 | 3 | 4 | 17 | 19 (2) | 19 |
| | | 19 | ♀ | 89·5 | 9 (2) | 9 (7) | 1 (1) | 5 | 0 | 0 | 1 | 1 | 14 | 17 (2) | 14 |
| | 11·6 | 20 | ♂ | 95·0 | 7 | 5 (3) | 1 (1) | 7 | 1 | 0 | 0 | 3 | 12 | 16 (1) | 17 |
| | | 19 | ♀ | 94·7 | 2 | 13 (2) | 2 (2) | 2 | 3 (2)† | 0 | 3 (1) | 1 | 10 | 17 | 16 (1)* |
| Control | 0 | 16 | ♂ | 6·25‡ | 0 | 0 | 0 | 0 | 0 | 0 | 0 | 0 | 0 | 0 | 0 |
| | | 14 | ♀ | 28·6‡ | 0 | 0 | 0 | 0 | 0 | 0 | 0 | 0 | 0 | 0 | 0 |

* Numbers of malignant tumours are in parentheses.

† Metastasis from other organs.

‡ Percentage of tumours located in organs other than those listed in the Table.

FIG. 1.—Cut surface of a hepatocellular carcinoma in CH after 39 weeks of treatment with 1/20 $LD_{50}$ DMN. The tumour tissue demonstrates pronounced haemorrhages. ×3.

almost equal survival times (Table I); these increased with decreasing dose levels. The frequency of the neoplasms did not differ among the various groups, although the animals in the lowest dosage groups received approximately 1/3 the amount of carcinogen until death as did those from the highest dosage groups.

### *Tumours resulting from chronic DEN treatment*

Following chronic DEN treatment up to 68% of the animals developed squamous cell papillomata or carcinomata of the maxilloturbinals and nasoturbinals, or of the maxillary sinuses (Table II). The largest number of these cases was found among the females in the highest and lowest dosage groups. In almost all treated animals the incidence of tumours in the area of the nasal cavities was greater amongst the females than the males. Ten, from a total of 78 nasal cavity tumours, penetrated the *lamina cribiformis* and invaded the brain (Fig. 2). The number of such malignant neoplasms increased with higher dosages. An even greater percentage of the animals demonstrated tumours in the region of the oesophagus and forestomach. These were always multiple tumours (in the forestomach up to 50 papillomata: Fig. 3) and when situated in the oesophagus obstructed the lumen. All tumours of the oesophagus were squamous cell papillomata with pronounced keratinization. Of the 102 animals with tumours of the forestomach, six had squamous cell carcinomata. Papillomata also occurred in the tongue, palate and pharynx. These were often extremely large and blocked the lumen of the pharynx and oesophagus. As a result several animals died through suffocation caused by a regurgitation of their feed. A large proportion of the hamsters also had aspiration pneumonitis. Tumours of the larynx, trachea and lungs were significantly fewer than those of the digestive tract (Table II). The occurrence of these two latter tumours was uniformly dispersed throughout all

FIG. 2.—Head of CH demonstrating a large tumour of the nasal cavities. The neoplasm has destroyed the temporal bone and thus shows exophytic growth. 18 weeks of treatment with 1/5 $LD_{50}$ DEN. ×3.

FIG. 3.—Forestomach of CH after 20 weeks of DEN treatment (1/10 $LD_{50}$): multiple papillomata of varying size have developed.

FIG. 4.—Hepatocellular carcinoma in CH after 25 weeks of treatment with 1/20 $LD_{50}$ of DEN. The tumour tissue on the left is poorly differentiated, whereas the tumour cells which have invaded the blood vessel on the lower right, are well differentiated. H. and E. ×40.

FIG. 5.—Pulmonary metastasis from the liver tumour in Fig. 3: well differentiated hepatic cells are situated in a pulmonary blood vessel and are within alveoli. H. and E. ×100.

dosage groups. Of eight animals with pulmonary tumours, three were diagnosed as adenocarcinomata, the remainder being adenomata. More than 70% of animals from all groups developed hepatic neoplasms. However, in only one instance was a hepatocellular carcinoma with metastasis to the pulmonary and renal vessels detected (Fig. 4 and 5). All other tumours were multiple hepatomata. The survival time of the hamsters increased with falling dose levels.

*Controls*

From a total of 40 female controls, three animals developed tumours; two in the uterus (1 adenocarcinoma, 1 myoma), one in the pancreas (an adenoma). From 40 male controls only one animal demonstrated a tumour (a fibrosarcoma of the epididymis). The life span of all tumour-bearing animals was longer than 80 weeks from commencement of treatment.

### DISCUSSION

When comparing the rates of DMN-induced tumours in the European, Syrian golden and Chinese hamsters it becomes apparent that the CH develops the greatest number of liver neoplasms in all dosage groups. The SGH also demonstrated a relatively high incidence of liver cell carcinomata, cholangiomata and haemangioendotheliomata. However, in contrast to the CH they showed a dose response relationship (Haas *et al.*, 1973; Tomatis *et al.*, 1964). The lowest incidence of such tumours was found in the EH, and again a dose response relationship existed. Furthermore, in the CH no differences were found between the tumour rates of the two sexes. Only one female developed a pulmonary metastasis with hepatic haemangioendothelioma characteristics. In contrast to these findings, the SGH (Herrold, 1967) and the EH (Mohr *et al.*, 1974*a*) demonstrated a markedly higher percentage of pulmonary metastases. A further difference in the reaction to DMN, as compared to the other two species, is the uniformity of tumours developed by the CH. Animals treated with 1/20 $LD_{50}$, which represents only 1/3 of the total dose received by those animals injected with 1/5 $LD_{50}$, exhibited a higher tumour incidence than those treated with the higher dose, this increased tumour incidence being dependent upon the longer life span of the animals. Neoplasms of other sites occurred in only three cases and all were adenocarcinomata of the nasal cavity. Renal tumours, which were induced at a relatively high rate in the EH (Mohr *et al.*, 1974*a*) were completely lacking in the CH.

Administration of DEN to the CH resulted in the induction of squamous cell carcinomata in the nasal and paranasal cavities, tongue, pharynx, oesophagus and forestomach. The squamous epithelium-coated organs of the digestive tract, oesophagus and forestomach, were the most frequently affected areas with only scanty neoplastic growth in the trachea. In contrast, the SGH and EH reacted to this nitroso-compound by the production of mainly neoplasms in the respiratory tract (Dontenwill and Mohr, 1961; Mohr *et al.*, 1972; Dontenwill, 1968). The nasal cavities were the main target organ in the EH (Mohr *et al.*, 1972); whereas in the SGH, DEN showed its highest carcinogenicity in the trachea (Dontenwill, 1968; Althoff *et al.*, 1971*b*). Noteworthy was the large number of hepatomata, which developed in the CH after DEN application. A comparably strong effect of DEN upon the liver has not been reported for the SGH or the EH.

The present results demonstrate that the CH, although belonging to the same family (Cricetidae), reacts in a markedly different manner from the other two species when treated with these two nitroso-compounds (DMN, DEN), the main target organs being the liver for DMN, and the digestive tract for DEN.

The authors are grateful to Christine Murphy for her assistance with the manuscript.

### REFERENCES

ALTHOFF, J. (1974) Simultaneous Tumours in the Respiratory System and Urinary Bladder of Syrian Golden Hamsters. *Z. Krebsforsch.*, **82**, 153.

ALTHOFF, J., KRÜGER, F. W., MOHR, U. & SCHMÄHL, D. (1971*a*) Dibutylnitrosamine Carcinogenesis in Syrian Golden and Chinese Hamsters. *Proc. Soc. exp. Med.*, **136**, 168.

ALTHOFF, J., WILSON, R. & MOHR, U. (1971*b*) Diethylnitrosamine Induced Alterations in the Tracheobronchial System of Syrian Golden Hamsters. *J. natn. Cancer Inst.*, **46**, 1067.

ALTHOFF, J., MOHR, U., PAGE, N. & REZNIK, G. (1974*a*) Carcinogenic Effect of Dibutylnitrosamine in European Hamsters (*Cricetus cricetus*). *J. natn. Cancer Inst.*, **53**, 795.

ALTHOFF, J., WILSON, R., CARDESA, A. & POUR, P. (1974*b*) Comparative Studies of Neoplastic Response to a Single Dose of Nitroso Compounds. *Z. Krebsforsch.*, **81**, 251.

DONTENWILL, W. (1968) Experimental Studies on the Organotropic Effect of Nitrosamines in the Respiratory Tract. *Food Cosmet. Toxicol.*, **6**, 571.

DONTENWILL, W. & MOHR, U. (1961) Carcinome des Respirationstraktes nach Behandlung von Goldhamstern mit Diäthylnitrosamin. *Z. Krebsforsch.*, **64**, 305.

HAAS, H., MOHR, U. & KRÜGER, F. W. (1973) Comparative Studies with Different Doses of N-Nitrosomorpholine, N-Nitrosopiperidine, N-Nitrosomethylurea and Dimethylnitrosamine in Syrian Golden Hamsters. *J. natn. Cancer Inst.*, **51**, 1295.

HERROLD, K. M. (1967) Histogenesis of Malignant Liver Tumors Induced by Dimethylnitrosamine. An Experimental Study in Syrian Hamsters. *J. natn. Cancer Inst.*, **39**, 1099.

MOHR, U., ALTHOFF, J. & PAGE, N. (1972) Tumors in the Respiratory System Induced in the Common European Hamster by N-Diethylnitrosamine. *J. natn. Cancer Inst.*, **49**, 595.

MOHR, U., HAAS, H. & HILFRICH, J. (1974*a*) The Carcinogenic Effects of Dimethylnitrosamine and Nitrosomethylurea in European Hamsters (*Cricetus cricetus* L.). *Br. J. Cancer*, **29**, 359.

MOHR, U., REZNIK, G. & REZNIK-SCHÜLLER, H. (1974*b*) Carcinogenic Effects of N-Nitrosomorpholine and N-Nitrosopiperidine on European Hamsters (*Cricetus cricetus* L.). *J. natn. Cancer Inst.*, **53**, 231.

MONTESANO, R. & SAFFIOTTI, U. (1968) Carcinogenic Response of the Respiratory Tract of Syrian Golden Hamsters to Different Doses of Diethylnitrosamine. *Cancer Res.*, **28**, 2197.

TOMATIS, L., MAGEE, P. N. & SHUBIK, P. (1964) Induction of Liver Tumours in the Syrian Golden Hamster by Feeding Dimethylnitrosamine. *J. natn. Cancer Inst.*, **33**, 341.

WEIL, C. S. (1952) Table for Convenient Calculations of Effective Dose ($LD_{50}$ or $ED_{50}$) and Instructions in their Use. *Biometrics*, **8**, 249.